# Simple Sequence Repeats and Mucoid Conversion: Biased *mucA* Mutagenesis in Mismatch Repair-Deficient *Pseudomonas aeruginosa*

**DOI:** 10.1371/journal.pone.0008203

**Published:** 2009-12-07

**Authors:** Alejandro J. Moyano, Andrea M. Smania

**Affiliations:** Centro de Investigaciones en Química Biológica de Córdoba (CIQUIBIC), CONICET, Departamento de Química Biológica, Facultad de Ciencias Químicas, Universidad Nacional de Córdoba, Córdoba, Argentina; Baylor College of Medicine, United States of America

## Abstract

In *Pseudomonas aeruginosa*, conversion to the mucoid phenotype marks the onset of an irreversible state of the infection in Cystic Fibrosis (CF) patients. The main pathway for mucoid conversion is mutagenesis of the *mucA* gene, frequently due to −1 bp deletions in a simple sequence repeat (SSR) of 5 Gs (G^5^-SSR_426_). We have recently observed that this *mucA* mutation is particularly accentuated in Mismatch Repair System (MRS)-deficient cells grown *in vitro*. Interestingly, previous reports have shown a high prevalence of hypermutable MRS-deficient strains occurring naturally in CF chronic lung infections. Here, we used *mucA* as a forward mutation model to systematically evaluate the role of G^5^-SSR_426_ in conversion to mucoidy in a MRS-deficient background, with this being the first analysis combining SSR-dependent localized hypermutability and the acquisition of a particular virulence/persistence trait in *P. aeruginosa*. In this study, *mucA* alleles were engineered with different contents of G:C SSRs, and tested for their effect on the mucoid conversion frequency and *mucA* mutational spectra in a *mutS*-deficient strain of *P. aeruginosa*. Importantly, deletion of G^5^-SSR_426_ severely reduced the emergence frequency of mucoid variants, with no preferential site of mutagenesis within *mucA*. Moreover, although mutagenesis in *mucA* was not totally removed, this was no longer the main pathway for mucoid conversion, suggesting that G^5^-SSR_426_ biased mutations towards *mucA*. Mutagenesis in *mucA* was restored by the addition of a new SSR (C^6^-SSR_431_), and even synergistically increased when G^5^-SSR_426_ and C^6^-SSR_431_ were present simultaneously, with the *mucA* mutations being restricted to −1 bp deletions within any of both G:C SSRs. These results confirm a critical role for G^5^-SSR_426_ enhancing the mutagenic process of *mucA* in MRS-deficient cells, and shed light on another mechanism, the SSR- localized hypermutability, contributing to mucoid conversion in *P. aeruginosa*.

## Introduction

Mutation is an essential feature of pathogenic prokaryotes, being involved in the generation of genetic variability and the acquisition of adaptive phenotypes. In fact, many virulence traits important in host colonization and sudden environmental changes (i.e., antimicrobial therapy, host immune response), are acquired by mutagenic events. Thus, factors which regulate mutagenesis may play critical roles in the pathogen's establishment and evolution within the host. Occasionally, increasing the mutation rate may facilitate the adaptation to different stimuli in bacterial populations [1]. Reports of this phenomenon involve different mechanisms, which include the presence of naturally-occurring stable mutators (i.e., DNA Repair-deficient strains), inducible or transient hypermutators (i.e., induction of error-prone DNA polymerases), and hypermutable genetic sequences (i.e., DNA simple sequence repeats [SSRs]). Regarding this last mechanism, SSRs are defined as tandem repetitions of short motives and are largely accounted for as a source of genetic variability [2], [3] through the generally accepted slipped-strand mispairing mechanism [2], [4]. In fact, there is extensive literature involving SSRs in pathogenesis of several bacterial species, frequently playing crucial roles in the antigenic- or phase-variation that occur at contingency loci (reviewed in [2]).

*Pseudomonas aeruginosa* is an opportunistic pathogen that chronically infects the lungs and airways of Cystic Fibrosis (CF) patients, which in order to persist in the CF lung, undergoes a genetic adaptation based on mutagenic events [5]. However, although the participation of stable hypermutators in this process has been investigated [6], [7], there are no reports about the role of SSR-localized hypermutability in the acquisition of phenotypes that allow its long-term persistence. Among these phenotypes, conversion to mucoidy (exopolysaccharide alginate-overproduction) is one of the most important virulence traits in *P. aeruginosa*, since it confers protection against the host immune response [8], [9], reactive oxygen intermediates [10], and pulmonary clearance [11]. In fact, the emergence of the mucoid phenotype in the CF lung marks the onset of an irreversible state of the infection and poor prognosis for the patient [12].

More than one possible pathway leading to alginate overproduction has been described [13], i.e. positive regulation by *rpoN* [14] or mutations in regulatory genes such as *mucB* and *mucD* [15]–[17]. However, the most frequent pathway that leads to the mucoid phenotype is the acquisition of loss-of-function mutations in a single gene, *mucA*, which encodes for a negative regulator of alginate production [18]. Studies of CF mucoid isolates have shown that *mucA* harbored loss-of-function mutations in more than 85% of isolates [11], [16], [19]. Similarly, work in our laboratory and by other researchers has shown *mucA* to be the main target for mutagenesis in mucoid variants obtained *in vitro* [20], [21]. These studies found that for several types of mutations, one of the most represented was the −1 deletion in a monomeric SSR of five Gs (G^5^-SSR_426_) located at position 426 from the *mucA* start codon (widely known as *mucA22* allele) [11], [16], [19]–[22].

In a previous recent work, we determined *in vitro* that two factors involved in the regulation of the overall mutation rate, MutS (a main component of the Mismatch Repair System) and Pol IV (the error-prone DNA polymerase encoded by *dinB*), were essential to establish *mucA* as the main target for mutagenesis in mucoid conversion, with these two factors having a prominent role in the generation of the *mucA22* allele [21]. Questions that still remained unsolved are: 1) why was there such a high percentage of mucoid isolates in which mutations in *mucA* were found? 2) what is special about *mucA* that makes it the main pathway to mucoid conversion (thus leaving a secondary role to other genes whose inactivation are also known to induce mucoidy, such as *mucB* and *mucD*)? 3) does *mucA* contain a hotspot for mutagenesis? 4) what is the role of G^5^-SSR_426_ in this phenomenon? Concerning this last question, since no study to date has evaluated the role of any SSR in *P. aeruginosa*, little is known about their relevance in this mutagenic adaptive processes. In an attempt to shed light on this, we designed and constructed *mucA* alleles with different SSR compositions by site directed mutagenesis, and then analyzed the emergence frequency of mucoid variants and the spectrum of *mucA* mutations in strains carrying the different *mucA* alleles. Assays were performed using a DNA Mismatch Repair System (MRS)-defective *mutS* strain for several reasons: 1) the low spontaneous rate of mucoid conversion and the low yield of *mucA22* alleles in nonmutator strains do not allow an accurate analysis in this experimental system [21]; 2) MRS-deficient strains most directly reflect the mutagenesis (in frequency and nature) of the ongoing DNA synthesis [23]; 3) they provide a larger yield of mucoid variants [21]; 4) this yield is enriched in *mucA22* alleles [21];. Furthermore, previous studies have reported a large proportion of *P. aeruginosa* hypermutator MRS-deficient strains occurring naturally in CF chronic infections [7], which has been proposed to catalyze the genetic adaptation for persistence in the CF lung environment [6]. This leads to the idea that the coexistence of SSRs and MRS deficiency might be a typical phenomenon in the CF lung.

In this work, we show that in a MRS-deficient background, G^5^-SSR_426_ was an essential hotspot biasing mutations to *mucA* thereby contributing, together with stable hypermutability, in the determination of *mucA* as the main pathway for mucoid conversion in *P. aeruginosa*.

## Results

### The Presence of SSRs in *mucA* Increases the Yield of Mucoid Variants in *P. aeruginosa*

**Figure 1:**
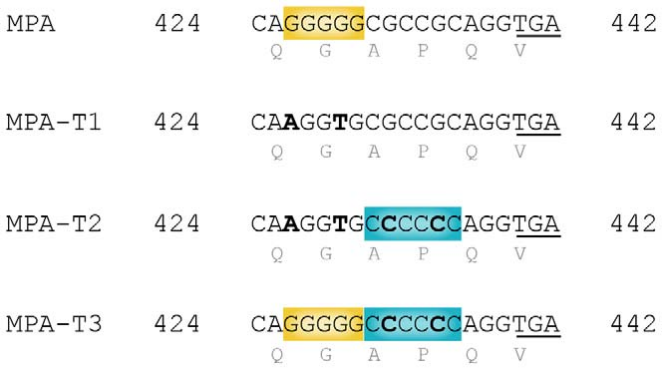
Site directed mutagenesis on the *mucA* gene. A fragment of the *P. aeruginosa mucA* gene where the site directed mutagenesis was performed is shown. Base changes (bold face) were designed in order to maintain the amino acidic sequence unaltered (shown below), and codons were chosen that are commonly used by *P. aeruginosa*. The premature stop codon generated by a hypothetical −1 bp deletion between positions 426 and 436 of *mucA* is underlined. Wild type *mucA* allele from MPA strain with G^5^SSR_426_ is highlighted in yellow. The *mucAT1* allele from strain MPA-T1 was generated by replacing G-to-A at 426 and G-to-T at 429, thus eliminating G^5^SSR_426_. The *mucAT2* allele from strain MPA-T2 was generated by replacing G-to-A at 426 and G-to-T at 429 (eliminating G^5^SSR_426_), and G-to-C at 432 and 435 to generate C^6^SSR_431_ (highlighted in blue). The *mucAT3* allele from strain MPA-T3 was generated by replacing G-to-C at 432 and 435 to generate C^6^SSR_431_ (highlighted in blue), and maintaining G^5^SSR_426_ (highlighted in yellow) unaltered.

As mentioned above, mutations in the *mucA* gene are known to be the major cause of mucoid conversion in *P. aeruginosa* [18]. Previous *mucA* sequence analyzes of mucoid isolates, obtained from CF patients as well as under laboratory conditions, showed that they mostly harbored the *mucA22* allele (a −1 bp deletion in a homopolymeric G:C SSR here referred to as G^5^-SSR_426_) [11], [16], [19]–[22]. In order to determine the role of G^5^-SSR_426_ in *mucA* mutagenesis leading to *P. aeruginosa* mucoid conversion, we constructed strain MPA-T1 with its *mucA* sequence lacking G^5^-SSR_426_ (*mucAT1* allele) (Figure 1). This strain was generated in a *mutS* deficient background in order to increase the yield of mucoid variants, and also because this background allows the direct observation of replicative errors without interference by the MRS.

Previous studies have established that mucoidy, a phenotype that is almost exclusively observed in chronic infections, could be reproducibly obtained *in vitro* from the effluent run-offs of continuous flow-cultured biofilms [20], [21]. Based on these antecedents, bacteria were grown in continuous cultured biofilms, with the effluents run-off being plated in order to score for mucoid variants (easily distinguishable as “mucous droplet-like” colonies). In addition, the mucoid phenotype of each variant was confirmed by the carbazole method which showed a≥3 fold increase in alginate production respect to the parental strain.

**Figure 2:**
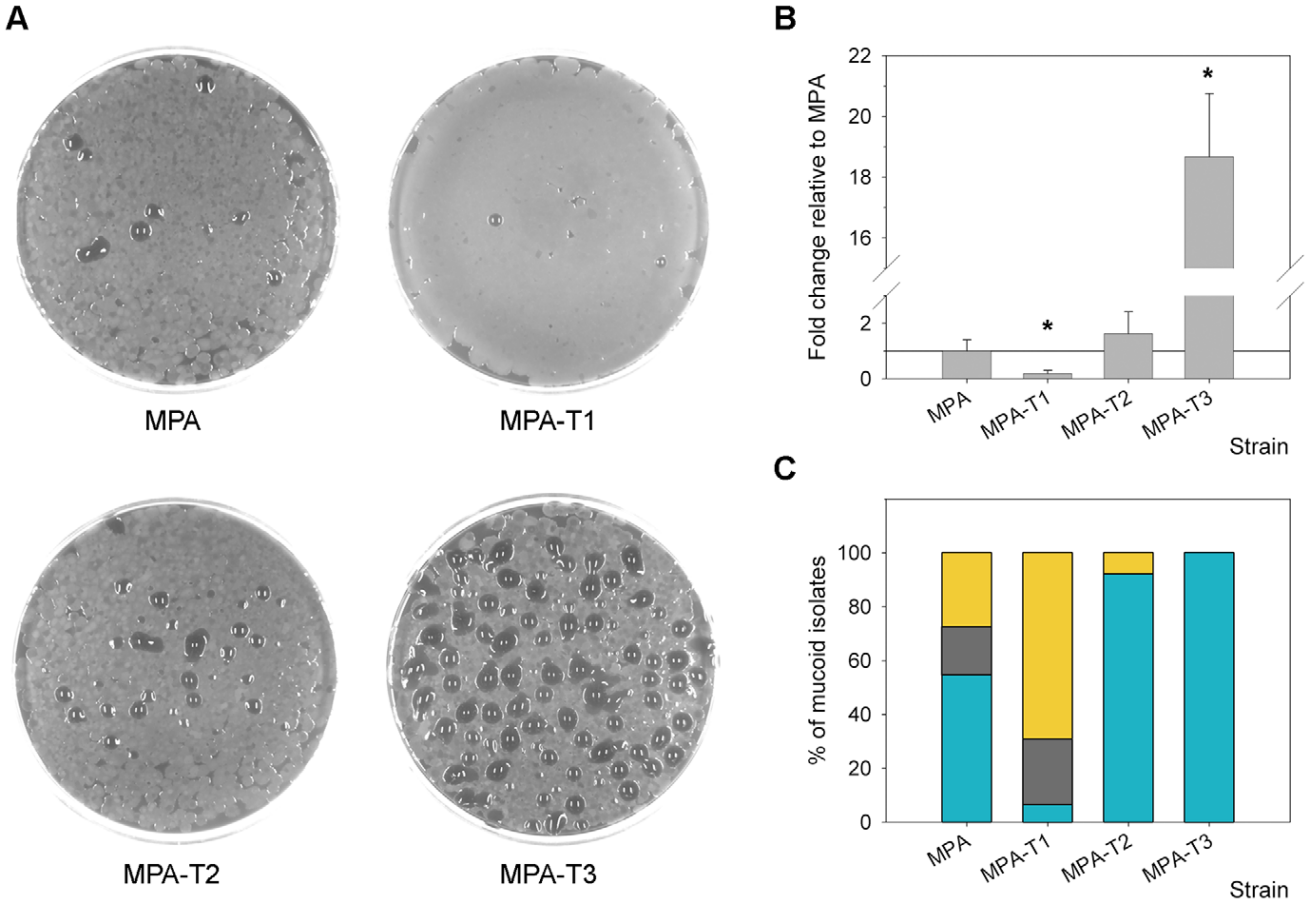
Role of SSRs in *P. aeruginosa* mucoid conversion. (A) Mucoid variants, visualized as mucous droplet-like colonies emerging from MPA, MPA-T1, MPA-T2 and MPA-T3 strains plated onto MMA plates. (B) Relative emergence of mucoid variants obtained from MPA-T1, MPA-T2 and MPA-T3 strains. The values were compared to those obtained for the MPA strain. *Values for MPA-T1 and MPA-T3 were significantly different (p<0.05, as determined by the t-test) from that of MPA. (C) Spectrum of *mucA* mutations observed in mucoid variants obtained from MPA, MPA-T1, MPA-T2 and MPA-T3 strains. Bars indicate the observed percentage for each kind of mutation of the total number of mucoid clones analyzed in each strain (total clones analyzed: MPA, 11; MPA-T1, 13; MPA-T2, 13; and MPA-T3, 15). Bars in yellow indicate those mucoid variants that occurred in the absence of *mucA* alterations. The observed mutations included base substitutions (gray) and −1 bp deletions in mononucleotide G:C SSRs (blue).

Then, the mucoid variant emergence frequencies of strains MPA-T1 and its parental MPA were compared. As shown in Figures 2A and 2B, the emergence frequency of mucoid variants suffered a significant 5.3 fold decrease (p<0.05, t-test) in strain MPA-T1 (0.09±0.05%) respect to strain MPA which carried the wild type *mucA* (0.48±0.20%), providing evidence that under the conditions used, G^5^-SSR_426_ was involved in the mucoid conversion process.

To test whether the elevated frequency of mucoid conversion was an exclusive feature of G^5^-SSR_426_, and to examine whether a SSR (different from G^5^-SSR_426_) was able to restore the level of generation of mucoid variants lost in MPA-T1, we tested strain MPA-T2, which lacked G^5^-SSR_426_ in *mucA*, but carried a new stretch of 6 Cs (C^6^-SSR_431_) located immediately after the region previously occupied by G^5^-SSR_426_ (Figure 1). It is worth noting that the hypothetical deletion of one C in C^6^-SSR_431_ generates the same premature stop codon as the *mucA22* allele. This last issue is important, in order to try to avoid as much as possible an altered emergence frequency of mucoid variants due to differential selection instead of differential mutagenesis between *mucA* alleles.

As shown in Figures 2A and 2B, the emergence frequency of mucoid variants in strain MPA-T2 (0.78±0.38%) showed an increase of 1.6-fold respect to MPA, which although not statistically significant (p>0.05, t-test) implied a 7.1-fold increase respect to MPA-T1. This result not only supports the previous observation that the presence of a SSR such as G^5^-SSR_426_ was determinant in the generation of mucoid variants, but also indicates that this function could be replaced by another SSR different from the wild type G^5^-SSR_426_.

In order to test the simultaneous effect of G^5^-SSR_426_ and C^6^-SSR_431_ on the emergence frequency of mucoid variants, a new strain was generated (MPA-T3) which carried both SSRs (G^5^C^6^SSR_426_) (*mucA-T3* allele) (Figure 1). In the same way, a hypothetical −1 deletion in G^5^C^6^SSR_426_ generates the same premature stop codon at 440 as the *mucA22* allele.

Strikingly, strain MPA-T3 showed a significant increase in the emergence frequency of mucoid variants of more than one order of magnitude (8.96±1.00%) respect to strains MPA or MPA-T2 (p<0.05, t-test) (Figure 2A and 2B). This result clearly indicates that the combination of both SSRs had a synergistic incremental effect on the yield of mucoid variants, as in the case of a larger single SSR.

To check that no variation in the global mutagenesis of the cell was acquired during strain constructions, the four strains (MPA, MPA-T1, MPA-T2 and MPA-T3) were subjected to the Rf-resistance test, which is a widely known assay to determine the overall mutagenic state of the cell [24]. The frequencies of Rf-resistant cells presented the expected values for *mutS*-deficient hypermutator strains, which were similar in the four strains (4.4-5×10^−6^ cells). Furthermore, in order to verify that the basal alginate production was not altered by the different *mucA* allele replacements, alginate production was measured in the four strains by the carbazole method [25]. Basal alginate production did not vary among strains MPA, MPA-T1, MPA-T2 and MPA-T3 (150–200 µg per ml of growth culture), which confirms that the modifications engineered in *mucA* did not produce a constitutive overproduction of alginate.

### SSRs Constitute Hotspots for Mutagenesis in *mucA*

In a recent study [21], we showed that the overall hypermutability generated by inactivation of the MRS gene *mutS* produced an increase in *mucA* mutagenesis that was accompanied by a decrease in the variability of mutations in *mucA*. For this *mutS* strain, mutagenesis in *mucA* was clearly the leading pathway for mucoid conversion, and interestingly, the most represented mutation was the −1 deletion in G^5^-SSR_426_ generating the *mucA22* allele [21]. In order to determine if the carriage of different *mucA* alleles affects the prevalence of the *mucA* conversion pathway, and to analyze the spectra of mutations of *mucA* alleles harboring different SSRs, the *mucA* gene of mucoid variants obtained from the four strains, MPA, MPA-T1, MPA-T2 and MPA-T3, was PCR amplified and subjected to automated direct sequence analysis.

Regarding the fraction of the mucoid isolates that harbored mutations in *mucA* in this study, MPA showed mutations in *mucA* for 73% of the mucoid isolates (Figure 2C). Analysis of *mucA* mutations demonstrated that 75% corresponded to the *mucA22* allele, with the remaining 25% being represented by C-to-T transitions (Table 1 and Figure 2C). Both observations about the prevalence and spectrum of *mucA* mutations are in agreement with our previous results [21], with this being the spectrum typically observed in MRS deficient strains [26], [27].

**Table 1:** Mutations in the *mucA* gene of mucoid isolates from strains MPA, MPA-T1, MPA-T2 and MPA-T3.

Strain and *mucA* mutation^a^	Stop^b^	Number of isolates
**MPA**		
ΔG at 426	TGA at 440	6
C→T at 424	TAG at 424	1
C→T at 436	TAG at 436	1
None	none	3
**MPA-T1**		
ΔC at 362	TGA at 385	1
G→A at 249	TGA at 247	1
C→T at 367	TAG at 367	1
C→T at 424	TAG at 424	1
C→T at 505	TAG at 505	1
none	none	9
**MPA-T2**		
ΔC at 431	TGA at 440	12
none	none	1
**MPA-T3**		
ΔG at 426	TGA at 440	5
ΔC at 431	TGA at 440	10

a ΔG at 426 corresponds to a −1 bp deletion within G^5^SSR_426_; ΔC at 362 corresponds to a −1 bp deletion within a mononucleotide SSR of four Cs from 362 to 365 (C^4^SSR_362_); ΔC at 431 corresponds to a −1 bp deletion within C^6^SSR_431_. “None” refers to conversion to mucoidy occurring in the absence of *mucA* mutations. The nature of these non-*mucA* alterations leading to a mucoid phenotype was not investigated in this work.

b Stop codon produced at the site of the mutation by substitutions or placed in frame by frameshift mutations.

Elimination of G^5^-SSR_426_ in MPA-T1 reduced the prevalence of *mucA* mutations among the mucoid isolates to 31% (Figure 2C). Notably, this result, together with the decrease in the emergence of mucoid variants in MPA-T1 (Figure 2A and 2B), suggest that after the elimination of G^5^-SSR_426_, mutagenesis of *mucA* was no longer the major pathway of mucoid conversion. The spectrum analysis of *mucA* also showed that no mutation had occurred in the region previously occupied by G^5^-SSR_426_. Instead, an increase in the variability of mutations was observed, in which four isolates (80%) harbored different C-to-T and G-to-A transitions, with one isolate (20%) having a deletion of one C in a stretch of four Cs located between 362 and 365 (C^4^SSR_362_) of the coding region of *mucA*, thus generating a premature stop codon at 385 (Table 1 and Figure 2C). This result demonstrates that in strain MPA-T1, mutagenesis in *mucA* was not biased to any particular DNA motif, indicating that there were no distinguishable hotspots for mutagenesis within the *mucA-T1* allele. Furthermore, this observation suggests that in strain MPA, the emergence of mucoid variants might not be due to the effect of scattered mutagenesis along the *mucA* gene and further selection, but could greatly depend on the presence of G^5^-SSR_426_ biasing mutations, thereby constituting a real hotspot for mutagenesis in a MRS-deficient background.

Notably in strain MPA-T2, which carried C^6^-SSR_431_, the proportion of mucoid variants that harbored mutations in *mucA* increased to 93% (Figure 2C), thus restoring and even surpassing the proportion observed for the MPA wild type allele. Interestingly, this strain showed a drastic reduction in the spectrum of *mucA* mutations, in which 100% were represented by a −1 bp deletion within C^6^-SSR_431_ (Table 1 and Figure 2C). Therefore, it is evident that this SSR (6 bp long) has a stronger biasing capacity respect to G^5^-SSR_426_ (5 bp long), which could be logically explained due to its larger size. However, as it has been previously observed in *E. coli* [28], the possibility that G:C SSRs may have different levels of mutagenesis depending on their orientations respect to the replication fork should also be considered (see Discussion).

In the case of MPA-T3, which carried G^5^C^6^-SSR_426_, 100% of the mucoid isolates harbored mutations in *mucA*, with these mutations being −1 bp deletions and showing a distribution between both SSRs of 67% for C^6^-SSR_431_ and 33% for G^5^-SSR_426_ (Table 1 and Figure 2C). This distribution of mutations is also consistent with the differential capacity of each SSR to bias mutations (Figure 2C), and could explain the previous observation of the higher mucoid emergence frequency for strain MPA-T2, although this was not statistically significant compared with MPA (Figure 2B). Furthermore, spectra observed in strains MPA-T2 and MPA-T3 clearly suggest that no other kind of alterations or rearrangements are occurring in the SSRs apart from the slipped-strand mispairing mechanism.

Taken together, these results indicate that G^5^-SSR_426_ is a hotspot, which, based on its length, is able to bias but not restrict mutations towards the *mucA* gene. Thus, G^5^-SSR_426_ constitutes a major element, without which, *mucA* ceases to be the leading pathway for conversion to mucoidy in MRS-deficient *P. aeruginosa*.

### Most of the Loss-of-Function Mutations Occur in the Periplasmic Coding Region of *mucA*, Independently of the SSR Content

We next performed a survey of different studies reporting mutations in the *mucA* gene from *P. aeruginosa* CF isolates, in order to analyze the way in which *mucA* is mutated. Four different studies [11], [16], [22], [29], along with own unpublished data, showed that the great majority of mucoid isolates obtained from CF are due to frameshifts or base substitutions along the coding sequence of *mucA* (Table 2). Among these mutations, different C-to-T transitions and −1 bp deletion frameshifts were the most represented, with most of these −1 bp deletions occurring in G^5^-SSR_426_, thus generating the *mucA22* allele (Table 2). As we observed for the mucoid variants obtained from MPA, MPA-T1, MPA-T2 and MPA-T3 (Table 1), and also for the clinical mucoid isolates, independent of the kind of mutation (either base substitution or −1 bp frameshift), in every study the great majority of *mucA* mutations (64–96%) generated a premature stop codon, thus providing a truncated version of MucA.

**Table 2:** Classification of *mucA* mutations reported in different studies for *P. aeruginosa* CF isolates.

Location	Analyzed isolates^a^	Kinds of mutations in *mucA* (%)				−1 bp in G^5^SSR_430_/Total −1 bp deletions (%)	Reference
		Transi-tions	Transver-sions	−1 bp deletions	Other indels^b^		
USA	41	34	9	32	25	67	[11]
Australia	22	50	0	29	21	57	[29]
Germany	14	41	0	53	6	88	[22]
Scandinavia	148	46	1	33	20	80	[16]
Argentina	24	29	8	63	0	67	Unpublished

a For each study, only the isolates for which the *mucA* mutations were reported were considered in the analysis.

b Insertion or deletion mutations different from −1 bp deletions.

It is important to point out that MucA is an anti-sigma factor located at the inner membrane of the cell, which has an N-terminal cytoplasmic region (MucA_7–57_), a transmembrane domain (MucA_84–104_) and a periplasmic C-terminal domain (MucA_113–170_) [30], [31]. Based on this protein domain structure, we then analyzed if the stop mutations were clustered at any particular region of *mucA*. With few variations among studies, stop mutations were concentrated almost exclusively in the periplasmic coding region of *mucA* (84–100%), in a proportion that was significantly higher than the few remaining stop mutations observed in the transmembrane (0–13%) (p<0.001, Z-test), and the cytoplasmic (0–3%) (p<0.001, Z-test) coding regions of *mucA*. Our own previous results with mucoid variants obtained from laboratory conditions showed a similar distribution of mutations along the *mucA* gene [21].

We next wondered whether the G^5^-SSR_426_ capacity of biasing mutations was a determinant factor of the region at which most mutations were found. As shown above, strains MPA, MPA-T2 and MPA-T3 (with their SSRs in the periplasmic coding region of *mucA*) had all the alterations affecting this domain (Table 1). In the case of strain MPA-T1, although the prevalence of *mucA* mutations was greatly decreased and demonstrated no defined hotspot for mutagenesis, most of the mutations found in *mucA* (80%) also affected the periplasmic coding region of the gene (Table 1). This domain distribution of mutations was not significantly different from that observed in clinical isolates (p = 0.989, Z-test), which indicates that the presence of G^5^-SSR_426_ does not play a major role in the selection of the functional domain of MucA in which mutations arise or are selected. Thus, other sequence properties of *mucA* and/or selection forces on the MucA domain that is mutated may prevail over the effect of any SSR producing a bias on mutagenesis.

In order to determine other properties in the sequence of *mucA*, which might lead to differential mutagenesis between domains, we further sought for all the possible positions at which premature stop codons could be generated by C-to-T transitions (which is the other large group of mutations observed in *mucA*). Thus, we surveyed all the CAG and CAA codons (which encode for Glutamine) and CGA (which encodes for Arginine), and their respective distributions in the *mucA* gene. Interestingly, MucA possesses 14 Glutamine residues, of which 12 (86%) are located within the periplasmic region, and the remaining 2 (14%) in the cytoplasmic region. No CGA codon for Arginine was observed in the whole coding sequence of *mucA*. If it is assumed that each of the 14 Glutamine codons possesses an equal or similar probability of changing to a stop codon, then the observation mentioned above suggests that this distributive bias of Glutamine codons towards the periplasmic coding region of *mucA*, might contribute to the higher prevalence of non-sense mutations within this domain. Nevertheless, since MucA is highly regulated by proteolysis in response to certain types of stress [31]–[36], this analysis cannot rule out the possibility of selection pressures favoring periplasmic over non-periplasmic truncated versions of MucA.

### G^5^-SSR_426_ Is Conserved in Most of the *P. aeruginosa* Strains, but Not in Other Pseudomonad Related Species

Since MucA is an anti-sigma factor highly conserved among several bacterial species (also known as RseA), we next scored for G^5^-SSR_426_ or a similar SSR in the *mucA* gene of other Pseudomonad related species [37], some of which are also known to have the possibility of acquiring a mucoid phenotype [38], [39]. The survey of *P. fluorescens* Pf-5, *P. putida* KT2440, *P. stutzeri* A1501, *P. syringae* 1448A and DC3000, *P. entomophila* L48, and *P. mendocina* ymp, revealed that although their identity scores respect to *P. aeruginosa mucA* sequence are high (≥72), none of these species harbored G^5^-SSR_426_ or any other G:C SSR exceeding 4 bp in their *mucA* genes, which indicates that G^5^-SSR_426_ might be exclusive for *P. aeruginosa*. Related to this, we then analyzed if G^5^-SSR_426_ was conserved intraspecifically by comparing the *mucA* sequence of several *P. aeruginosa* strains. This survey was performed on an own collection of 38 CF strains (not shown), one environmental strain Hex1T [40], and strains PAO1, PA14, LESB58 and PA7, whose genome sequence data are available online [37]. A comparative analysis showed that the scores of the identities of the *mucA* gene of the different strains respect to the *mucA* sequence of PAO1were ≥98. Thus, it was not surprising to find that G^5^-SSR_426_ was conserved in the *mucA* sequences of almost every strain of *P. aeruginosa*. However, G^5^-SSR_426_ was not present in strain PA7. Instead, PA7 showed an intriguing feature: a new SSR, also of five Gs located at 354 (G^5^-SSR_354_) in place of the SSR of four Gs observed in every other strain of *P. aeruginosa*. Curiously, in strain PA7 a −1 bp deletion within G^5^-SSR_354_ generates the same premature stop codon at 440 as that produced by a −1 bp deletion within G^5^-SSR_426_ of the other *P. aeruginosa* strains. Thus, the presence of G^5^-SSR_426_ and/or G^5^-SSR_354_,which showed to be absent in every other Pseudomonad species here analyzed, seems to be an intraspecifically highly conserved feature, which is so far unique for *P. aeruginosa*.

## Discussion

In *P. aeruginosa*, mutagenesis in the *mucA* gene is the main pathway for conversion to mucoidy, an alginate-overproducing phenotype most associated with chronic infections in the airways of CF patients. Mucoid conversion dramatically increases the resistance of the bacteria to pulmonary clearance mechanisms, and marks the transition to an irreversible state of the infection [12], [13]. In a previous recent work, we show that the mutagenic activity of the error-prone DNA polymerase Pol IV and the MRS loss-of-function, major factors involved in the inducible and stable hypermutability of the cell respectively, are key determinants targeting *mucA* for mucoid conversion *in vitro* [21]. Under a MutS deficiency and Pol IV proficiency background, the spectrum of mutation in *mucA* is dominated by frameshift mutations in a particular mononucleotide G:C SSR of five Gs (here denominated as G^5^SSR_426_), suggesting that instability of this SSR could be another main determinant which leads to mucoid conversion and makes *mucA* a very attractive model to study the involvement of the mutagenic mechanisms in *P. aeruginosa* adaptation.

Thus, in the present study, we attempted to elucidate the role of G^5^SSR_426_ in the process of conversion to mucoidy in a *mutS* deficient strain, which is to our knowledge the first systematic study of the mutagenic role of a SSR in *P. aeruginosa*. We used the *mucA* gene and allelic variants of *mucA*, engineered to contain different G:C SSR compositions (Figure 1), as a forward system for the detection of mutations that confer mucoidy. Importantly, although the system designed in this work for *P. aeruginosa* lacks the benefit of selection, it shares the advantage of other chromosomal forward systems used in other bacterial species for the detection of broad mutational spectra [41], [42] over reversion systems, in which the number of sites and kinds of mutations that can produce the revertant phenotype are quite limited [43]–[48].

As mentioned above, mutagenesis in *mucA* constitutes the main pathway for conversion to mucoidy, although other possible pathways that convergently lead to alginate overproduction have also been described [14], [15], [17]. Therefore, *mucA* mutations have previously been considered to be “pathoadaptive”, since their occurrence and the subsequent action of natural selection shift bacterial adaptation to the pathologic lung environment [49]. In the present study, we demonstrate in a MRS-deficient strain that mutagenesis in *mucA* is not scattered, but is in fact biased towards the −1 bp deletion in G^5^SSR_426_. Related to this, we show that the elimination of G^5^SSR_426_ not only significantly reduced the mutation frequency in *mucA* (Figures 2A and 2B), but also expanded the spectrum of mutations with no apparent hotspot (Table 1 and Figure 2C). Most importantly, in the absence of G^5^SSR_426_, *mucA* was no longer the major pathway for mucoid conversion, providing strong evidence that G^5^SSR_426_ makes *mucA* more prone to mutation. Interestingly, analysis of the coding sequences of the *mucB* and *mucD* genes (whose inactivation also lead to mucoid conversion) revealed that whereas *mucD* lacks mononucleotide G:C SSRs exceeding 4 bp, *mucB* possesses a SSR of 5 Cs. However, a recent study on a large collection of CF isolates which reported several mutations for the *mucB* gene showed that none of these occurred within this C-SSR [16]. This observation results intriguing since this C-SSR and G^5^SSR_426_ in *mucA* which could be considered comparable SSRs, are actually not. This allows the speculation that not only the preferential *mucA* mutagenesis may have a major selective component to it, but also that the C-SSR in *mucB* does not represent a hotspot for mutagenesis within the gene, as it has been observed for G^5^SSR_426_ in *mucA*.

Furthermore, taking into account the results on *mucA* mutagenesis obtained in our previous work, it seems that G^5^SSR_426_ is a hot substrate for both Pol IV-induced replication errors and correction by mismatch machinery [21]. Thus, our model of *mucA* mutagenesis demonstrates the combined action of the three mechanisms known to increase cellular mutability: inducible hypermutability, stable hypermutability and SSR-localized hypermutability. Also, previous studies have reported a high proportion of *P. aeruginosa* hypermutator MRS-deficient strains naturally occurring in CF pulmonary chronic infections [7], with SOS induction of Pol IV being recently described in *P. aeruginosa* [50], [51]. All these observations suggest that stressful environmental lung conditions might be propitious for mucoid conversion via MutS, Pol IV and G^5^SSR_426_-dependent mutagenesis. In this context, it is important to remember that approximately 85% of the *P. aeruginosa* mucoid clinical isolates harbor mutations in *mucA*, and that for 25–40% of these mutations, −1 bp deletions are found in G^5^SSR_426_ [11], [16], [19], [22]. In this way, given that longer SSRs (as experimentally confirmed with *mucA* alleles carrying C^6^SSR_431_ or G^5^C^6^SSR_426_) may confer a level of hypermutability that could turn *mucA* the exclusive genetic pathway to mucoid conversion, we conclude that not only the existence, but also the length of G^5^SSR_426_, is a major determinant of the *mucA*-dependent mucoid conversion process.

Regarding the different level of mutagenesis observed between G^5^SSR_426_ and C^6^SSR_431_, it should be considered that not only their length, but also their orientations respect to the replication fork may influence the mutagenesis outcome. In fact, it has been observed in *E. coli*, that frameshift mutagenesis on G:C SSRs displays an asymmetry during leading and lagging-strand replication [28]. Accordingly, a strand bias has also been reported for the mutagenic activity of Pol IV [52], which has been shown to be the main DNA polymerase involved in mutagenesis of G^5^SSR_426_ *in vitro* [21]. In this sense, further studies are necessary to elucidate the effect of strand biased mutagenesis in *P. aeruginosa*.

Furthermore, it results disturbing that the increase in the mutation frequency observed due to C^6^SSR_431_- and G^5^C^6^SSR_426_-localized mutagenesis, was produced in strains that were already stable hypermutators. As these hypermutator strains have been frequently reported for CF patients [7], our results indicate that under such a hypermutable background, genes involved in virulence which possess large G:C SSRs should deserve a special concern.

On the other hand, the survey of different studies which reported the mutations in the *mucA* gene observed in *P. aeruginosa* CF isolates, showed that the great majority of the mutations in *mucA* generate premature stop codons at its periplasmic domain coding region, with most of these mutations being −1 bp deletions within G^5^SSR_426_ or different C-to-T transitions (Table 2). This way of periplasmic mutagenesis has also been observed in mucoid variants emerged *in vitro*, from hypermutator and non-mutator strains of *P. aeruginosa* [20], [21]. Here we observed that elimination of G^5^SSR_426_ did not change the functional domain at which most *mucA* mutations were found (Table 1). This means that other sequence features of *mucA* and/or selective forces favoring periplasmic mutated versions of *mucA* might play a role in the distribution of mutations observed along the gene. As a possible explanation, we observed that the periplasmic, but not the transmembrane/cytoplasmic domains of MucA, is rich in Glutamine, with this being the substrate for the generation of premature stop codons by C-to-T transitions. Considering that the C-to-T transition is the most frequent kind of mutation, together with the −1 bp deletion in G^5^SSR_426_, this Glutamine codon distribution might contribute to the generation of stop codons at the periplasmic domain of *mucA*. Nevertheless, MucA is a protein which is highly and differentially regulated by proteolysis at its periplasmic, transmembrane and cytoplasmic domains [31]–[36]. As an example, it has been recently observed that in a mucoid isolate, overexpression of truncated *mucA* alleles at levels of saturation of the proteolytic enzymes capacities, reverts the phenotype to a non-mucoid state [31]. In this sense, *mucA* alleles mutated at its periplasmic region, might still retain some degree of regulatory properties on alginate production or other processes. Thus, differential selective pressures could exist favoring some truncated versions of MucA over others. Related to this, it would be interesting to observe if the addition of a new G:C SSR in the cytoplasmic or transmembrane domains could determine a biased mutagnesis towards them.

Summing up, in this work we present another mechanism, SSR-localized mutagenesis, which might act together with inducible and stable hypermutability enhancing mutagenesis in *mucA*. Thus, these results contribute to the understanding of the mutagenic process which leads to mucoid conversion in *P. aeruginosa*, one of the hallmarks of chronic infection in the airways of CF patients.

## Materials and Methods

### Bacterial Strains, Plasmids and Culture Media

The bacterial strains and plasmids used in this study are described in Table 3. *P. aeruginosa* MPA strain [53] was kindly provided by Dr Michael Jacobs from the University of Washington Genome Center (USA). Transposon insertions within the *mutS* gene were confirmed by PCR analysis following the provider's instructions, and the resulting hypermutable phenotype was confirmed by the rifampin resistance test (see below). To prepare inocula, bacteria were routinely cultured on LB (1% NaCl, 1% soy peptone and 0.5% yeast extract) agar plates from frozen stocks and subcultured in LB liquid medium overnight at 37°C with shaking at 250 r.p.m.

**Table 3:** Bacterial strains, plasmids and oligonucleotides used in this study.

Strains, vectors and primers	Description^a^	Source or Reference
**Strains**		
*P. aeruginosa*		
MPA	*mutS::*IS*lacZ*A/hah-Tc^R^, MPAO1 derivative	[53]
MPA-T1	Tc^R^, MPA carrying a *mucAT1* allele	This study
MPA-T2	Tc^R^, MPA carrying a *mucAT2* allele	This study
MPA-T3	Tc^R^, MPA carrying a *mucAT3* allele	This study
*E. coli*		
XL1-Blue	host strain for DNA manipulation	[60]
SY327 λ*pir*	Rf^R^; recipient to propagate pKNG-101 derivatives	[61]
SM10 λ*pir*	Recipient for conjugal transfer of pKNG-101 derivatives	[61]
**Vectors**		
pGEM-T Easy	Ap^R^; PCR product cloning vector	Promega
pGEM-*mucAT1*	Ap^R^; *P. aeruginosa mucAT1* cloned in pGEM-T Easy	This study
pGEM-*mucAT2*	Ap^R^; *P. aeruginosa mucAT2* cloned in pGEM-T Easy	This study
pGEM-*mucAT3*	Ap^R^; *P. aeruginosa mucAT3* cloned in pGEM-T Easy	This study
pKNG101	Sm^R^, suicide delivery plasmid containing *sacB* gene (Suc^S^)	[55]
pKNG-*mucAT1*	Sm^R^, Suc^S^, pKNG101 carrying *mucAT1*	This study
pKNG-*mucAT2*	Sm^R^, Suc^S^, pKNG101 carrying *mucAT2*	This study
pKNG-*mucAT3*	Sm^R^, Suc^S^, pKNG101 carrying *mucAT3*	This study
**Primers**		
MucPA-F	5′-GAAGCCTGACACAGCGGCAAATGC-3′	[21]
MucPA-R	5′-CCTCAGCGGTTTTCCAGGCTGGCTGC-3′	[21]
MucBamHI-F	5′-TATGGATCCTGAAGCAATCGACAAAGCTC-3′	This study
MucXbaI-R	5′-TTATCTAGAAGCTGGGAGGGATCGAACTT-3′	This study
MucT1-F	5′-GCGAAGAGCAAGGTGCGCCGCAGG-3′	This study
MucT1-R	5′-CCTGCGGCGCACCTTGCTCTTCGC-3′	This study
MucT2-F	5′-GCAAGGTGCCCCCCAGGTGATCACCAACTCCTC-3′	This study
MucT2-R	5′-CTGGGGGGCACCTTGCTCTTCGCTGTAGCCGG-3′	This study
MucT3-F	5′-AGCAGGGGGCCCCCCAGGTGATCA-3′	This study
MucT3-R	5′-TGATCACCTGGGGGGCCCCCTGCT-3′	This study

a Resistance markers: Tc, tetracycline; Rf, rifampin; Sm, streptomycin; Ap, ampicillin; Suc, sucrose.

AB minimal medium [20] was used to grow continuous cultured biofilms. Mucoid maintenance agar (MMA) plates were used to score for mucoid colonies [54]. Antibiotics were used at the following concentrations unless otherwise indicated: ampicillin (Ap), 50 µg ml^−1^; streptomycin (Sm), 200 µg ml^−1^; rifampin (Rf), 100 µg ml^−1^.

### Construction of Strains MPA-T1, MPA-T2 and MPA-T3 by *mucA* Site Directed Mutagenesis

A set of different *mucA* alleles were engineered to eliminate G^5^-SSR_426_ or to contain different SSRs, by changing the specific codons for alternative codons (commonly used by *P. aeruginosa*) of the same amino acid and thus maintaining the primary structure of MucA intact (Figure 1).

To generate the strain MPA-T1, site directed mutagenesis in *mucA* was performed by replacing the endogenous *mucA* gene with a fragment containing a mutated version of *mucA* that lacked G^5^-SSR_426_ (*mucAT1*), with codon CAG (Gln_142_) changed to codon CAA, and codon GGG (Gly_143_) changed to codon GGT (Figure 1). The fragment was generated by PCR overlapping extension as follow: first, a PCR product was amplified using primers MucBamHI-F (containing an engineered *BamH*I site) and MucT1-R (containing the codon substitutions) with genomic DNA from MPA as the template. Simultaneously, a second PCR product was obtained using primers MucT1-F (which overlaps with MucT1-R) and MucXbaI-R (with an engineered *Xba*I site). Both resulting PCR products were gel purified (Qiagen) and ∼60 ng of these products were combined for a second PCR reaction which began with three cycles in the absence of added primers and was followed by 30 cycles with the addition of primers MucBamHI-F and MucXbaI-R. The resulting 1014 bp PCR fragment containing allele *mucAT1* was cloned into pGEM-T Easy (Promega) and then subcloned into the BamHI-XbaI restriction sites of the suicide vector pKNG101 [55] in order to generate pKNG-*mucAT1*.

For the generation of strain MPA-T2, the endogenous *mucA* was replaced with the *mucAT2* allele. Plasmid pKNG-*mucAT2* was constructed following the same steps as pKNG-*mucAT1*, except that the allele *mucAT2* was designed with the following codon substitutions: CAG to CAA (Gln_142_); GGG to GGT (Gly_143_); GCG to GCC (Ala_144_); and, CCG to CCC (Pro_145_) (Figure 1). The fragment containing allele *mucAT2* was generated with the overlapping primers MucT2-F and MucT2-R (containing the codon substitutions) and the common primers MucBamHI-F and MucXbaI-R, as described above.

The generation of MPA-T3 was assessed following the same previous steps, except that the overlapping primers MucT3-F and MucT3-R (containing the codon substitutions) were used. In this case, codon substitutions for the generation of *mucAT3* consisted in GCG to GCC (Ala_144_) and CCG to CCC (Pro_145_) (Figure 1).

The resulting pKNG101 derivatives were maintained in *E. coli* SY327 λ*pir* before being transferred to *E. coli* SM10 λ*pir* to perform biparental mating with *P. aeruginosa* MPA and to carry out allelic exchange following standard protocols [56]. Briefly, mating cells were plated on LB agar plates containing 200 µg ml^−1^ Sm to select a single homologous recombination event with 50 µg ml^−1^ Ap to counterselect the donor *E. coli*. Then, Sm-resistant *P. aeruginosa* transconjugants that showed sucrose sensitivity were grown overnight in LB medium. Finally, the second recombination event was selected by plating on LB agar supplemented with 16% sucrose and scoring for Sm-sensitive clones. Allelic exchange was confirmed by DNA sequencing analysis.

### Bacterial Growth in Continuous Cultured Biofilms

Bacteria were grown in continuous-flow culture PVC tubing (USP class VI) with an inner diameter of 1.6 mm. Briefly, ∼10^8^ cells from overnight (ON) cultures were resuspended in 1 ml of AB medium before being injected upstream of the tubing and incubated for 1 h without flow to allow bacterial attachment to the substratum. Then, the flow was resumed and biofilms were constantly irrigated with AB medium at 0.05 ml min^−1^ for five days. Mucoid colonies were scored by visual inspection after plating adequate aliquots of the run-off effluents on MMA agar plates, before incubating them at 37°C for 48 h and again for five days at room temperature [21].

All determinations were carried out at least in triplicate for three independent experiments.

### Determination of the Mutation Frequency

Single colonies of each strain were cultured overnight in LB medium at 37°C for 24 h with shaking at 250 r.p.m. Appropriate dilutions of the cultures were plated on LB agar plates to determine the total number of viable cells, or on LB agar supplemented with 100 µg ml^−1^ Rf to count the number of rifampin-resistant cells, following incubation overnight at 37°C. Then, the mutation frequency was determined as the ratio of the number of rifampin–resistant cells and the number of viable cells. Determinations were carried out in duplicate for three independent experiments, and the results were expressed as means with their standard deviations.

### Determination of Alginate Production

Bacteria were grown in LB medium supplemented with 2% glycerol at 37°C for 72 h with shaking at 250 r.p.m. After incubation, the cultures were centrifuged at 7000 g for 15 min and the alginate present in the supernatants was precipitated with 3 vols of ethanol at −70°C for 24 h, followed by centrifugation at 18000 *g* for 15 min. The pellet was resuspended in water and the alginate was quantified by the carbazole assay [25], using alginate (Sigma) and glucuronic acid as standards.

### PCR Amplification and DNA Direct Sequencing of the *mucA* Gene

The primers MucPA-F and MucPA-R used to amplify the coding region of the *mucA* gene are described in Table 3. Colony PCR of the *P. aeruginosa* MPA, MPA-T1, MPA-T2 and MPA-T3 strains, as well as of their respective mucoid derivates, was performed as described previously [21]. The PCR products were extracted from agarose gels with a Gel purification kit (Fermentas) and subjected to automated direct DNA sequence analyzes (CRC-DNA Sequencing Facility, Univ. of Chicago, USA) by using the primers described above. To identify mutations in the *mucA* gene, the sequences obtained from the different mucoid variants were analyzed for homologies with the *mucA* (PA0763) sequence annotated in the *P. aeruginosa* Genome Project [57], by using the BLAST software [58].

### Data Resources and Software

Sequences of the *mucA* (*rseA*) gene from *P. aeruginosa* strains PAO1 (PA0763),PA14 (PA14_54420), LESB58 (PALES_45801), PA7 (PSPA7_4756) and from the Pseudomonad species *P. fluorescens* Pf-5 (PFL_1449), *P. putida* KT2440 (PP_1428), *P. syringae* phaseolicola 1448A (PSPPH_3954), *P. syringae* pv. tomato str. DC3000 (PSPTO_4223), *P. stutzeri* A1501 (PST_1224), *P. entomophila* L48 (PSEEN4295), and *P. mendocina* ymp (Pmen_1468) were downloaded from the *Pseudomonas* Genome Database [37] (http://www.pseudomonas.com). Alignments were obtained using the Clustal W software [59].

### Statistical Analysis

Statistical analysis of the data was performed with the two-tailed t-test using the GraphPad Instat software, and with the Z-test (normal approximation) using the Primer of Biostatistics software. Differences were considered statistically significant when p<0.05.
